# EV-Planner: a machine learning approach to electric vehicle charging infrastructure planning

**DOI:** 10.3389/frai.2026.1742351

**Published:** 2026-04-07

**Authors:** Bharath Anand, Ananth Grama

**Affiliations:** 1 Elmore Family School of Electrical and Computer Engineering, Purdue University, West Lafayette, IN, United States; 2 Department of Computer Science, Purdue University, West Lafayette, IN, United States

**Keywords:** charging stations, clustering, electric vehicles, hyperparameter, multi-objective, neural network, suggest-accept clustering

## Abstract

Electric Vehicles (EVs) offer pathways to lower emissions and increased energy efficiency. However, a broad and equitable adoption of electric vehicles can only be realized with well-planned growth in public charging infrastructure. We propose EV-Planner, a software tool that formulates EV charging station placement as a constrained multi-objective optimization problem and computes approximate solutions to these problems. In contrast to previous efforts, EV-Planner considers multiple criteria, including minimizing average user-to-station distance, maximizing fairness in coverage, and balancing load across stations. We propose a clustering-based approximation to solve the constrained multi-objective problem, along with a Suggest-Accept (SA) criterion that iterates over two steps: (i) generation of suitable clusters; and (ii) acceptance of a subset of clusters based on the specified cost function and constraints. To tune the hyperparameters in SA clustering, we develop a neural network model and an associated training procedure. Based on real-world data from 10 US states, EV-Planner reduces average distances to charging stations by 52.3%, the number of users who are more than three miles from a charging station by up to 10.7 times, and overloaded EV stations by 71.7% over current baselines, offering a promising solution to the problem of planning EV infrastructure. The code is available at https://github.com/bharathanand0/EV-Planner.

## 1 Introduction

The US EPA estimates that transportation accounted for ~28% of emissions in 2022, with the vast majority coming from road transportation (U.S. EPA, 2024). Electric vehicles (EVs) offer a promising pathway to reducing transportation emissions and costs (Intergovernmental Panel on Climate Change, 2022), and many countries have aggressive targets for growth in EV adoption (BloombergNEF, 2023). However, meeting these targets will require significant growth in public charging infrastructure. It is estimated that the US alone will require 1.2 million new public charging stations—over 20 × the current number—by 2030 to support its goals for EV adoption (Bauer et al., 2021).

Charging station placement is naturally a multi-objective optimization problem. Some key considerations that must be factored in are minimizing the average distance from users to their nearest charging station, load-balancing (balancing the number of users per station) across charging stations, and ensuring that everyone in the target region has access to a charging station within a certain distance. In current practice, charging stations are constructed in an *ad hoc*, market-driven manner, leading to significantly sub-optimal locations, as we show in our results. Recognizing the need for a systematic approach, prior work has proposed methods for charging station placement based on mathematical programming, game theory and machine learning (Abbasi et al., 2024; Alanazi et al., 2023; Bose et al., 2023; Parent et al., 2024; Hosseini and MirHassani, 2019; Lin and Hua, 2015; Lam et al., 2013; Padmanabhan et al., 2021; Pawar et al., 2022; von Wahl et al., 2022; Xiong et al., 2021, 2018); however, these efforts do not adequately address the constrained multi-objective nature of the problem.

We present EV-Planner, a tool for placing EV charging stations using unsupervised machine learning. We first formulate a constrained multi-objective optimization problem that captures considerations such as user convenience, capacity, and coverage metrics. Recognizing the computational hardness of the problem, we propose an approximate solution technique based on clustering, augmented with a suggest-accept (SA) heuristic for constraint satisfaction. Our solution is an iterative two-step approach that consists of a cluster generation step, where the points are clustered using an existing clustering algorithm, followed by a cluster acceptance step, where user-specified cost functions and constraints are imposed. This process accepts a subset of clusters and a subset of points in each cluster, leaving the remaining points for subsequent iterations. To optimize the various hyperparameters associated with the suggest-accept heuristic, we propose a computationally efficient neural network-based approach for hyperparameter selection. We use EV-Planner to locate charging stations for 10 US states based on publicly available geospatial datasets. Our results indicate that EV-Planner greatly improves on current charging station locations. The proposed SA clustering method outperforms existing clustering algorithms such as k-means (Lloyd, 1982), bisecting k-means (Steinbach et al., 2000), and constrained k-means (Bennett et al., 2000).

### 1.1 Related work

**Figure 1:**
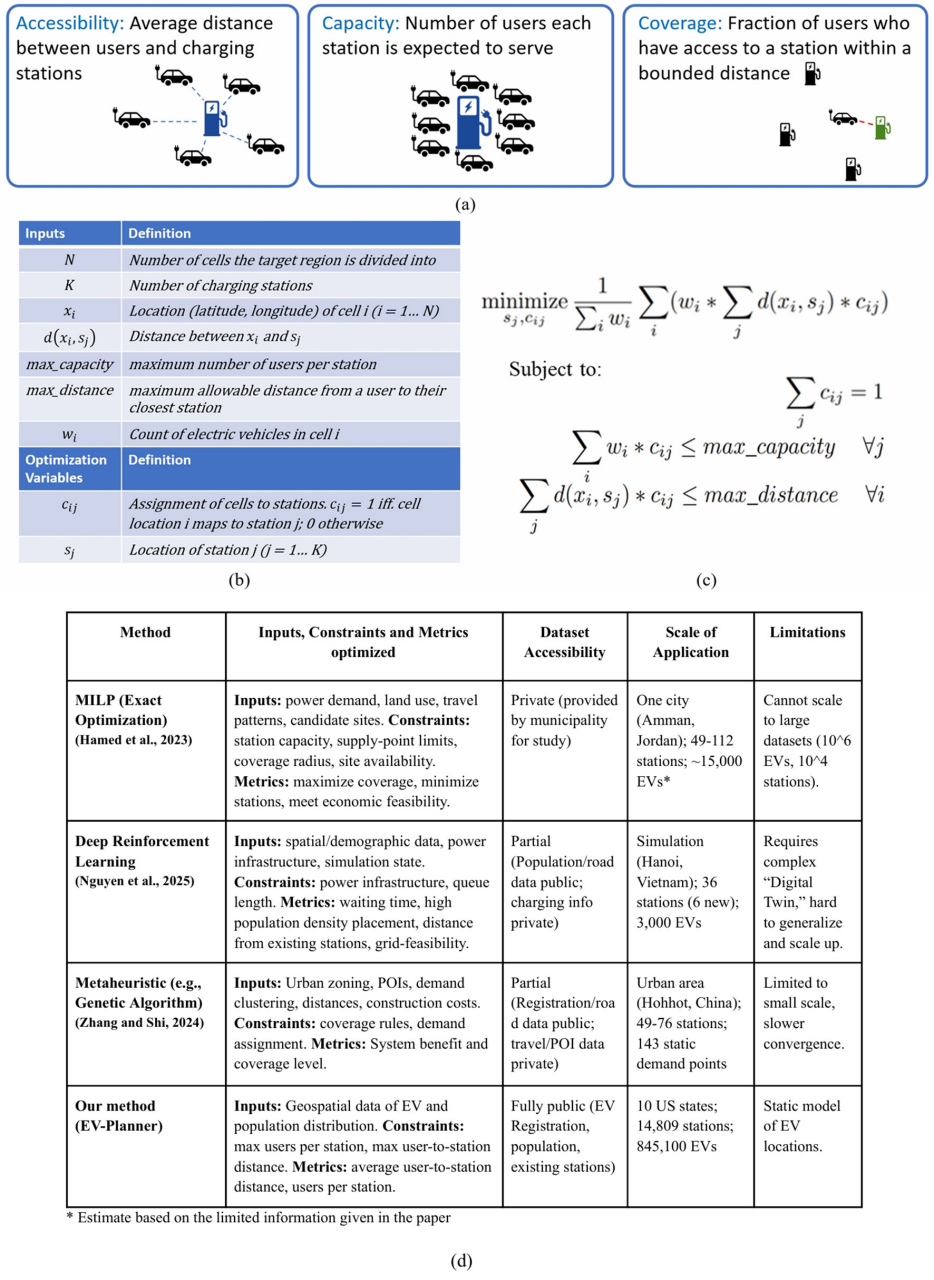
**(a)** Metrics for optimizing EV charging station placement, **(b)** Variable and constant definitions, **(c)** optimization formulation, and **(d)** Comparison of EV-Planner to other methods.

The problem of EV charging station placement has attracted significant attention in multiple disciplines. Various approaches have been proposed, ranging from optimization-based methods to data-driven techniques. Figure 1d shows a comparison of EV-Planner to recent related work (Nguyen et al., 2025; Zhang and Shi, 2024; Hamed et al., 2023) in various aspects such as input, constraints, metrics optimized, dataset availability, and scale of application. We also categorize the existing literature into three main groups: (i) mathematical programming approaches; (ii) game-theoretic methods; and (iii) machine learning approaches.

#### 1.1.1 Mathematical programming

Many early approaches to charging station placement were based on mathematical programming techniques. (Lam et al. 2013), and (Hamed et al. 2023) formulated the problem as a mixed-integer linear program (MILP) with objectives to minimize metrics such as construction cost or total travel distance. (Parent et al. 2024) used MILP to balance user convenience with infrastructure costs, considering traffic flows and travel patterns. (Xiong et al. 2021) extended these models to incorporate temporal dynamics, accounting for variations in charging demand through the day. (Zhang and Shi 2024) used the NSGA-II metaheuristic to solve their multi-objective optimization problem.

(Lin and Hua 2015) adapted the flow-capturing location-allocation model to EV charging, aiming to maximize the interception of origin-destination flows. (Hosseini and MirHassani 2019) enhanced this approach by integrating traffic assignment models to capture the impact of charging station placement on route choices. While these mathematical programming approaches provide optimal solutions for small-scale problems, they are constrained by scalability to large-scale real-world scenarios.

#### 1.1.2 Game-theoretic methods

Game theory has served as a popular approach to charging station placement. (Abbasi et al. 2024) modeled the problem as a Stackelberg game between charging station operators and EV users, where operators make placement decisions anticipating user behavior. (Alanazi et al. 2023) employed a multi-leader-follower game to capture competition among multiple charging service providers. (Bose et al. 2023) integrated coalition formation games to analyze cooperative station placement among multiple stakeholders, showing that cooperation can lead to more efficient infrastructure deployment. While game-theoretic methods excel at capturing strategic interactions, they often make simplifying assumptions about user preferences, player rationality, and information availability.

#### 1.1.3 Machine learning

Researchers have applied reinforcement learning to optimize EV charging station placement, with the environment model simulating EV user behavior and charging patterns. Deep Q-networks have been used to learn optimal placement strategies that maximize service quality while minimizing infrastructure costs (Padmanabhan et al., 2021; von Wahl et al., 2022). Clustering-based approaches represent another recent direction in the literature. K-means clustering and density-based clustering methods like DBSCAN have been applied to identify potential charging station locations based on spatial distributions of EVs (Ameer et al., 2025; Sánchez et al., 2022). Hybrid methods that combine clustering with mathematical programming have also been explored (Zhang and Shi, 2024).

#### 1.1.4 Limitations of existing approaches and EV-Planner contributions

Despite the considerable literature on EV charging station placement, existing approaches face one or more of the following limitations, as summarized below and in Figure 1d.

- Computational scalability: mathematical programming approaches such as MINLP/MILP are not scalable to large problem instances, particularly at the state or national level.
- Lack of multi-objective consideration: many approaches focus on a single objective (e.g., minimizing distance between vehicles and stations) without adequately addressing multiple potentially competing objectives such as fairness, load balancing, and coverage.
- Data requirements: many sophisticated methods require detailed data on travel patterns and user behavior that may not be readily available.
- Constraint handling: existing approaches often struggle to incorporate hard constraints such as station capacity and maximum distance limits.

EV-Planner addresses these limitations by formulating the problem as a constrained multi-objective optimization problem and proposing Suggest-Accept (SA) Clustering, which effectively incorporates multiple constraints and objectives. We overcome the limitations of classical clustering methods that have hardwired objectives like cluster radius and limited ability to impose constraints. Furthermore, we propose a neural network-based hyperparameter selection method that further enhances the efficiency and effectiveness of SA Clustering in identifying solutions along the multi-objective Pareto frontier.

### 1.2 Metrics in EV charging station placement

In EV charging station placement, prior work has employed various metrics that capture different attributes of a desirable placement. We briefly discuss these metrics, since understanding them is crucial for comparison of different approaches.

#### 1.2.1 Accessibility metrics

Accessibility metrics focus on the ease with which users can reach charging stations. The most common accessibility metrics used in the literature are geographical distance and travel time. As explained in Section 2, EV-Planner uses a flexible approach to accommodate any of the above distance metrics. EV locations and charging station locations are represented as (latitude, longitude) coordinates, and any accessibility metric that can be expressed as a “distance" function between an EV location and a station location can be used in EV-Planner, including Euclidean distance, Haversine distance, road distance, and travel time. In our experiments, we utilize the Haversine distance metric for evaluating EV-Planner as well as all baselines.

#### 1.2.2 Coverage metrics

Coverage metrics assess the extent to which the population has access to charging services. Binary coverage models, where a user is considered “covered" if they are within a specified distance (see Section 1.2.1 for distance functions supported) of a charging station, are commonly used. Graduated coverage models, where the coverage value decreases with distance, have also been proposed. EV-Planner uses a binary coverage model, and incorporates coverage as a constraint by requiring all EV locations to be within a user-specified maximum distance of a charging station. The user of EV-Planner can vary this constraint, *e.g.*, to explore tradeoffs between coverage and the number of charging stations.

#### 1.2.3 Load balancing metrics

Balanced utilization of the charging infrastructure is crucial for system efficiency. Load balance is typically evaluated based on the distribution of the number of EVs assigned to each station. EV-Planner incorporates load balancing by constraining the maximum number of EVs assigned to each station. In our experiments, we evaluate the number of overloaded stations resulting from the solutions produced by EV-Planner and the baselines.

We focus our discussion on the metrics presented in Figure 1a. However, the EV-Planner framework is flexible and can be extended to incorporate other metrics such as land and construction costs as a function of charging station location, proximity to retail facilities, electric grid capacity, etc.

## 2 Methods

### 2.1 Constrained optimization formulation of EV charging station placement

The multitude of metrics involved in EV charging station placement motivates us to formulate the problem as a constrained optimization problem. This formulation allows some metrics to be specified as constraints while others can be specified as part of a cost function to be minimized.

The constrained optimization problem formulated by EV-Planner is presented in Figure 1. The inputs to the formulation (Figure 1b), which are provided by the user, are:

- Geospatial data providing the spatial distribution of EVs across the target region. Following common practice in geospatial datasets, we assume the target region is tessellated into a hexagonal grid of *N* cells. *x*_*i*_ is a (latitude, longitude) vector denoting the location of cell *i*, and *w*_*i*_ is the number of EVs in it. While EV-Planner can work with varying grid resolutions, the size of the optimization problem (number of inputs and variables) grows with finer resolution. In our work, we follow the H3 geospatial indexing system (Uber Technologies Inc., 2024) resolution 8, where each cell has an area of 0.74*km*^2^. We believe this resolution provides a good balance between solution precision and runtime.
- The number of charging stations to place (*K*). While any value can be used, an unrealistically low or high value can make the results less meaningful. For our experiments across 10 US states, we used the number of actual existing stations in each state at the time of this work (U.S. Department of Energy, 2024) as the value of *K* for EV-Planner and all baselines.
- User-specified constraints on the maximum number of EVs a station can serve (*max*_*capacity*) and the maximum allowable distance from an EV to a charging station (*max*_*distance*).
- A pre-defined distance function *d*(*x*_*i*_, *s*_*j*_) such as Haversine distance, road distance, or travel time.

The variables that are optimized by EV-Planner are:

- *s*_*j*_, which represents the locations of charging stations, and
- *c*_*ij*_, a set of *N*×*K* Boolean variables where *c*_*ij*_ is 1 if cell *i* is assigned to EV station *j* and 0 otherwise.

Each of the three metrics from Figure 1a can be incorporated into either the objective function or constraints within EV-Planner. For this work, we adopt the specific formulation shown in Figure 1c where the objective function minimizes average user-to-closest-station distance, while the constraints limit the maximum number of EVs assigned to any station and the maximum distance from any user to a station.

The formulation presented in Figure 1c is a mixed integer non-linear programming problem (Sahinidis, 2019; Belotti et al., 2013) since the product of the distance *d*(*x*_*i*_, *s*_*j*_) and user-to-station assignment *c*_*ij*_ appears in the objective function and maximum distance constraint. It can be linearized using well-known techniques that involve the addition of auxiliary variables and constraints (Asghari et al., 2022). However, both MINLP and MILP are NP-Hard, and hence using MINLP/ILP solvers is not feasible for our problem instances (hundreds of thousands of EVs and thousands of desired charging station locations).

We observe that EV charging station placement can be approximated by a clustering problem, since at its core, the objective is to associate users with nearby stations, much as points are associated with nearby cluster centers. Therefore, we investigate the use of clustering as a computationally efficient approximate solution. To address the gap between contemporary clustering algorithms and the needs of the constrained optimization problem we formulate, we propose a new clustering method called Suggest-Accept clustering.

### 2.2 EV-Planner

**Figure 2:**
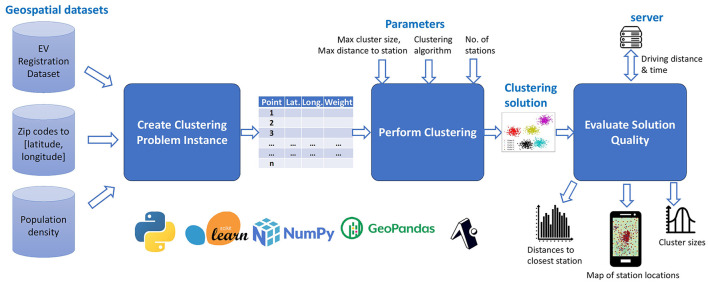
Overall flow of EV-Planner.

EV-Planner (Figure 2) is a software framework for computing the locations of electric vehicle charging stations that consists of three key steps: (i) creation of a clustering instance from the input data; (ii) performing clustering using a method that we propose called Suggest-Accept Clustering; and (iii) evaluating and visualizing the resulting solution. These steps are described in the following subsections.

#### 2.2.1 Creating a clustering instance

The first step within EV-Planner is the creation of a clustering problem instance from the input data. The inputs consist of geospatial data specifying (i) the number of EVs registered by ZIP code within the target region, (ii) the boundaries of each ZIP code, and (iii) the spatial distribution of population density within the target region.

To create a clustering instance, we tessellate the target region into a hexagonal grid at H3 resolution 8 (Uber Technologies Inc., 2024). We distribute the number of EVs in each ZIP code into the cells that fall within that ZIP code in proportion to the population density. This step is solely to increase the resolution of the EV data, and can be skipped when EV data is available at a finer resolution. We consider each of the *N* cells in the geospatial grid to be a point and the number of EVs in that cell (*w*_*i*_) to be the weight of the point. The desired number of charging stations (*K*) is specified as the number of clusters. In our work, we specify *K* to be equal to the number of existing charging stations in the target region for EV-Planner and all baselines so that we can evaluate how our methods improve upon existing station locations.

#### 2.2.2 Suggest-accept clustering

Clustering is an extensively studied field (Gan et al., 2020) with several algorithms and wide-ranging applications. Classical clustering algorithms implicitly minimize intra-cluster distance (*e.g*., the distance between a data point and its assigned centroid) to produce clusters that are as tight and distinct as possible. They do not naturally handle a multi-objective cost function with constraints distinct from the cost function. In our context, while the goal of minimizing user-to-station distance is naturally captured, the other two metrics (capacity and coverage) do not neatly fit into the ambit of classical clustering algorithms. Improvements of clustering algorithms have been proposed that try to produce uniformly sized clusters (Steinbach et al., 2000) or constrain cluster sizes (Bennett et al., 2000). However, as shown in our results, applying these algorithms (specifically, k-means, bisecting k-means, and constrained k-means) leaves significant room for improvement in the quest for high-quality solutions that satisfy all constraints. To address this challenge, we propose suggest-accept (SA) clustering, an iterative two-stage heuristic clustering method that can incorporate multiple objectives and constraints.

SA Clustering, outlined in Algorithm 1, takes as input a set of points *P* to be clustered, weights for each point (*W*), the target number of clusters *K*, as well as a user-specified cost function and constraints. SA Clustering iteratively executes two key steps—the suggest step and the accept step—until the required number of clusters have been created (lines 3–7 in Algorithm 1). In the suggest step (line 4), an existing clustering algorithm (k-means in our implementation) is used to cluster the remaining points. In the accept step (line 5), a subset of the clusters and a subset of the points within each cluster are accepted based on the degree to which they minimize the cost function while satisfying the constraints. The two-step process repeats until all points have been clustered. Convergence is guaranteed by ensuring that a minimum (non-zero) number of clusters is accepted in each iteration. Note that the term iteration refers to iterations of the suggest-accept steps and not iterations of the clustering algorithm used within the suggest step.

Algorithm 1 **Meta-training and meta-testing of TCPL.**

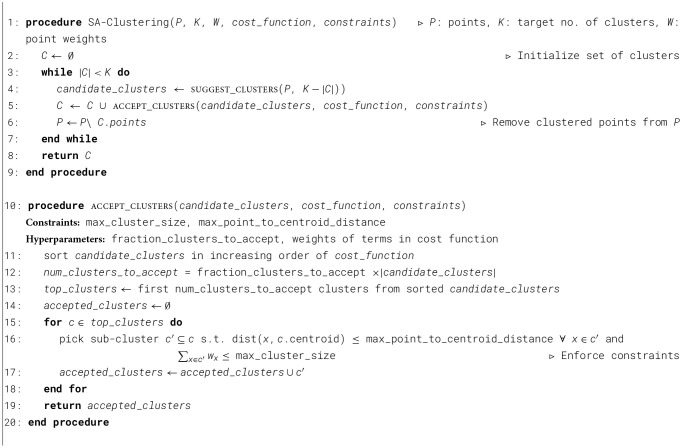

The details of the accept step are presented in function accept_clusters (lines 10–20 of Algorithm 1). The function receives as inputs the candidate clusters identified in the suggest step, as well as the cost function to be minimized and constraints. The cost function and constraints could incorporate any of the three metrics of the EV charging station placement problem, *viz*., accessibility, capacity, and coverage. In EV-Planner, we use a weighted sum of two terms in the cost function to evaluate a cluster: the mean point-to-centroid distance across all points in the cluster and the cluster size (sum of point weights). These terms are normalized to their largest values across all candidate clusters. The accept step first sorts candidate clusters in increasing order of the cost function (line 11). Next, the top candidate clusters to be accepted are identified based on the hyperparameter fraction_clusters_to_accept, the number of candidate clusters, and the sorted list of candidate clusters (lines 12–13). For each of these top clusters *c*, we pick a sub-cluster *c*′ (subset of points within the cluster) that satisfies both the max_cluster_size and max_point_to_centroid_distance constraints (line 16). The resulting sub-cluster is added to the list of accepted clusters (line 17), which is returned (line 19). Optionally, a third hyperparameter, relaxation_factor, may be used to soften the constraints in order to enable faster convergence (this is not included in Algorithm 1 for maintaining simplicity of explanation).

#### 2.2.3 Neural network based hyperparameter selection

The SA clustering algorithm uses the following key hyperparameters:

- The fraction of suggested clusters to accept in any given iteration (fraction_clusters_to_accept).
- The relative weights of different metrics in the cost function. In our case, the cost function has two terms (the normalized mean point-to-centroid distance across all points in the cluster and the normalized cluster size). We weight the first term by hyperparameter weight_distance and the second term by 1− weight_distance.
- The relaxation factor (relaxation_factor) to apply to soften the hard constraints in order to enable faster convergence.

Sweeping these three hyperparameters at a fine granularity, results in a large space (> 5,000,000 combinations), and running SA Clustering for each of these combinations would be prohibitively time-consuming. To address this challenge, we propose a neural network (NN) based hyperparameter selection method that efficiently identifies the Pareto frontier of the desired metrics. The method has the following steps:

1. We first sample the hyperparameter space by picking points that lie on a coarse-grained grid. In our experiments, hyperparameter fraction_clusters_to_accept was swept in steps of 0.05 in the range [0.1, 0.5]; hyperparameter weight_distance was swept in steps of 0.1 in the range [0.3, 1]; and hyperparameter relaxation_factor was swept in steps of 0.05 in the range [1, 1.5]. This results in a limited space of 792 hyperparameter combinations. We run SA Clustering on the input data with these hyperparameter combinations to compute the final metrics.
2. The dataset is split into train-validation-test subsets and used to train a regression NN to predict the performance of SA Clustering for a given hyperparameter combination. We use Neural Architecture Search (NAS) to search for the best NN architecture (in our experiments, we found that an MLP with four hidden layers and 40 neurons per layer consistently performed the best across all target regions; however, we note that the dataset on which the NN is trained, and hence the weights, are specific to each target region).
3. Finally, we sample a fine-grained uniform grid in the hyperparameter space. For each combination, we use the trained NN to predict the performance of SA Clustering (final clustering metrics) and create a predicted Pareto frontier. We take the hyperparameter combinations that lie along the predicted Pareto frontier and run SA Clustering to compute the actual clustering solutions.

Our results show that the NN-based hyperparameter selection method drastically cuts down on the runtime needed (by over 5,500 × compared to running SA Clustering on all hyperparameter combinations), while being able to accurately identify points on the Pareto frontier.

## 3 Results

We implemented EV-Planner in Python using several open-source libraries, including GeoPandas, numpy, and scikit-learn. We also developed a cross-platform Android/iOS mobile app using the Expo framework (Expo, 2024) that uses the Google Maps API to visualize the locations of charging stations suggested by EV-Planner and compare them to the locations of current charging stations.

We evaluated EV-Planner with data for 10 US states taken from the Atlas EV Hub (Atlas EV Hub, 2024), the US DoE Alternative Fuels Data Center (U.S. Department of Energy, 2024), and the Kontur geospatial data hub (Kontur Inc., 2024), which provide the geospatial distribution of EV registrations, the locations of current charging stations, and fine-grained population density distributions across the target states, respectively. The 10 states were selected to ensure that we had samples from each of the four quartiles in terms of both the number of EVs and the ratio of EVs to existing charging stations. Ensuring spread in the number of EVs allows us to evaluate the placement methods across different data scales, while spread in the EVs-to-stations ratio tests the methods with varying levels of station contention. For the NN-based hyperparameter selection, we created a training set of 792 points for each state, of which 25% were used for testing, with the remaining 75% being split in a 90:10 ratio for training and validation. The NN was implemented using the MLP regressor in scikit-learn, using the Adam optimizer, learning rate = 0.004, batch size = 32, momentum = 0.9. The mean squared error loss function was used for training, the evaluation metric was *R*^2^, and the regularization techniques used were weight decay = 0.0001 and early stopping.

All experiments were run on a workstation with an Intel Core i7-12700 CPU and 32GB RAM. We found that EV-Planner is quite efficient when applied at the state level (*e.g*., placing 3,771 stations for 171,000 EVs across the state of New York takes less than a minute).

For the following results, we used EV-Planner to place a number of charging stations equal to the current number of charging stations in each state (obtained from U.S. Department of Energy, 2024).

### 3.1 EV-Planner reduces average distances to charging stations

**Figure 3:**
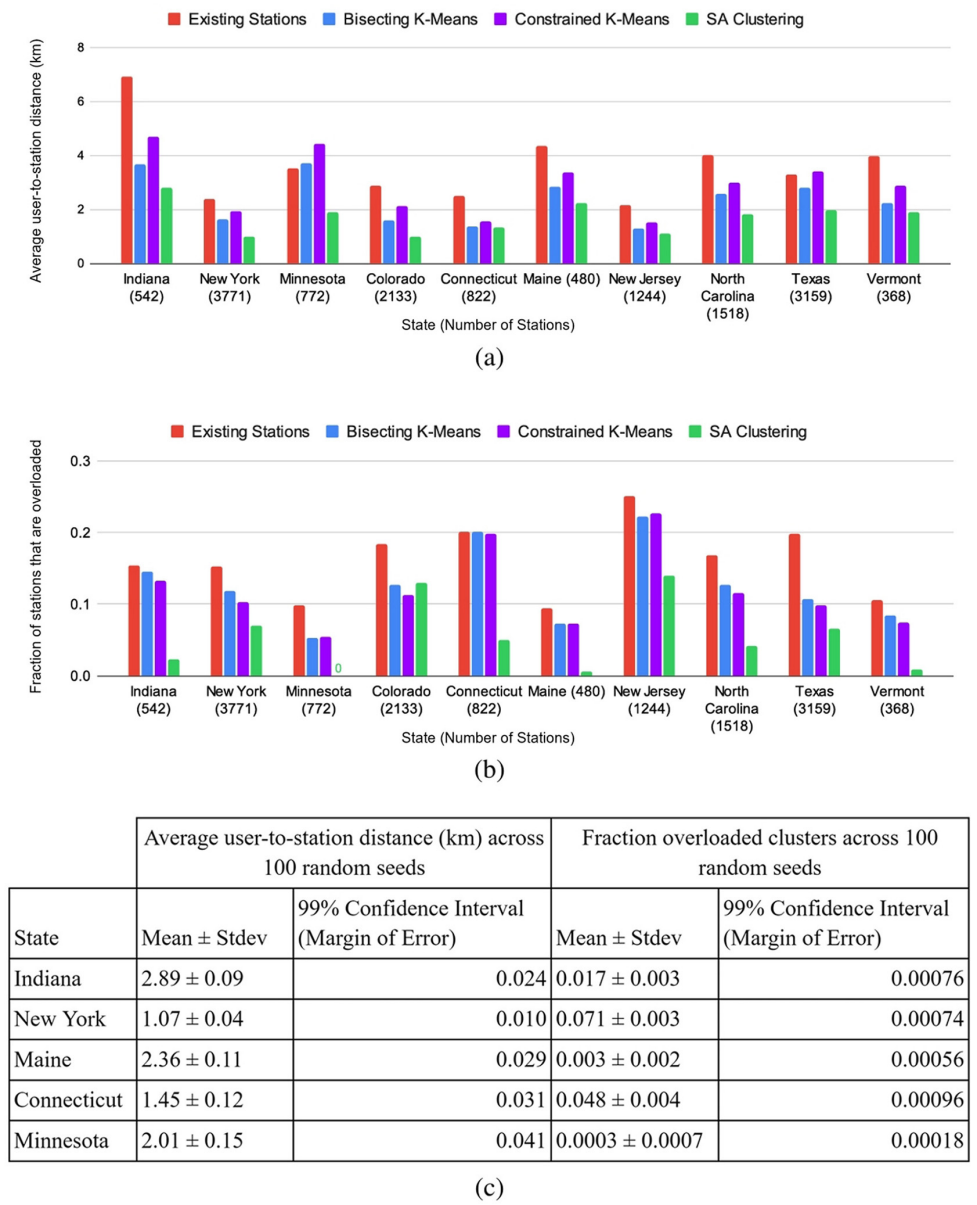
EV-Planner results compared to three baselines—existing station locations, Bisecting K-means, and Constrained K-means: **(a)** Average user-to-closest-station distance, and **(b)** Fraction of charging stations that are overloaded. **(c)** Statistical analysis of EV-Planner results across 100 random seeds.

Figure 3a presents the average user-to-closest-station distance for EV-Planner compared to three baselines—current actual station locations, bisecting k-means, and constrained k-means. EV-Planner consistently improves upon current locations by significant margins, with an average of 52.3% across all states. This amounts to an estimated savings of 106 million miles driven per year across EVs in these 10 states. EV-Planner also improves upon bisecting k-means and constrained k-means by 26.4 and 36.0%, respectively.

### 3.2 EV-Planner reduces the number of overloaded EV charging stations

Figure 3b presents the number of overloaded charging stations for solutions computed by EV-Planner compared to the current charging station locations, bisecting k-means, and constrained k-means. On average, EV-Planner improves upon current station locations by 71.7%, and upon bisecting k-means and constrained k-means by 62.3 and 59.2%, respectively. This result depends on the specific threshold for the number of EVs assigned to a station that would make it overloaded. For the results presented in Figure 3b, this threshold was set to 75. In our dataset, most states had roughly 20–50 times as many cars as EV stations. Hence, we experimented with various values of the threshold, ranging from 50 to 150, and observed consistent improvements over the baselines.

### 3.3 EV-Planner reduces the number of users who do not have access to a station within three miles by 10.7 fold

The improvements of EV-Planner are consistent across distance thresholds from 2 to 10 miles. Thus, EV-Planner can help ensure more equitable access to EV charging infrastructure.

### 3.4 Statistical validity

To ensure the statistical validity of our results, we ran EV-Planner with 100 random seeds. The table in Figure 3c presents the mean, standard deviation, and 99% confidence interval for average user-to-station distance and fraction of overloaded stations. We observe that both the standard deviation and 99% confidence interval are quite tight, suggesting the stability of EV-Planner and the proposed methods.

### 3.5 SA clustering outperforms other clustering algorithms

**Figure 4:**
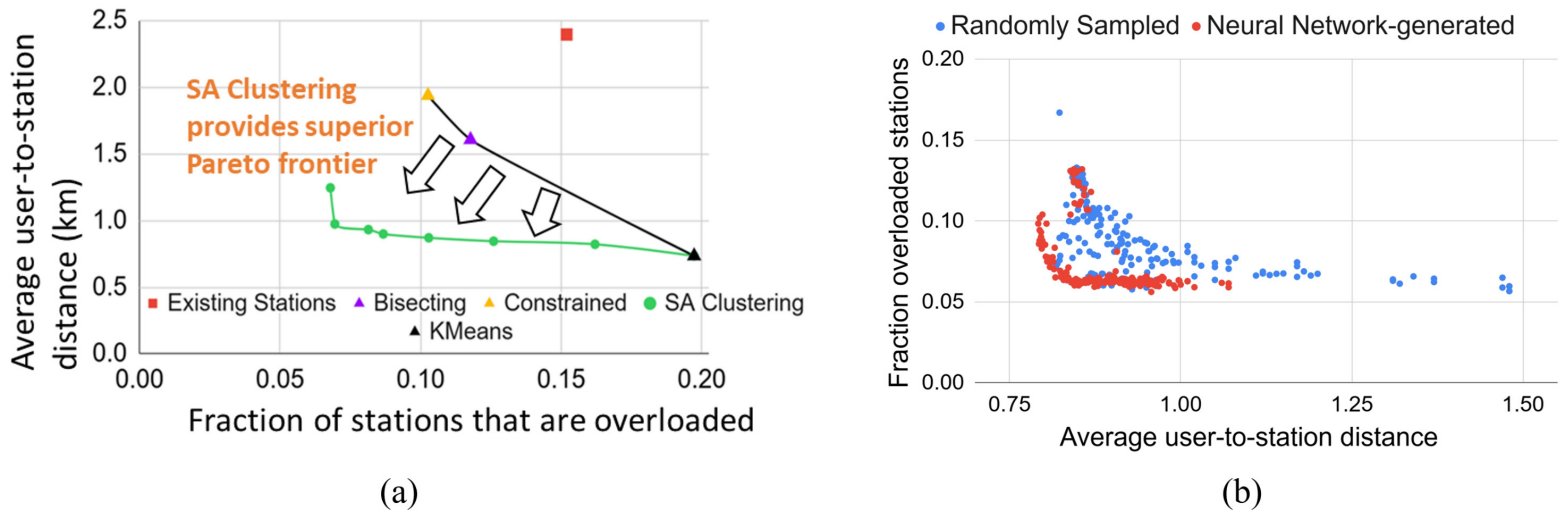
**(a)** Comparison of Pareto frontier achieved by SA Clustering with other clustering algorithms; **(b)** Efficacy of neural network-based hyperparameter selection in identifying the Pareto frontier.

Figure 4a presents a comparison of SA clustering with three existing clustering algorithms—k-means (Lloyd, 1982), bisecting k-means (Steinbach et al., 2000), and constrained k-means (Bennett et al., 2000)—on two metrics, namely the average user-to-station distance and the percentage of overloaded stations. The Pareto frontier achieved by SA clustering (by varying its hyperparameters) is clearly superior to the solutions provided by other clustering algorithms.

### 3.6 Efficacy of neural network-based hyperparameter selection

As shown in Figure 4b, NN-based hyperparameter selection is able to select hyperparameter values that result in solutions along the Pareto frontier much better than random sampling of the hyperparameter space.

### 4 Discussion and limitations

We presented EV-Planner, a software tool that formulates EV charging station placement as a constrained multi-objective optimization problem and computes approximate solutions using Suggest-Accept (SA) Clustering, a new clustering method that we propose. We demonstrate that EV-Planner is able to determine station locations at scale—across 10 US states with over 845,000 vehicles and over 14,800 charging stations, while cutting down on average user-to-station distance and overloaded charging stations. We identify limitations and directions for building upon this work. First, we believe that we have demonstrated our methods on sufficiently large-scale datasets and observed improvements across all 10 states considered. Nevertheless, our methods could be applied to additional regions to further establish their broad applicability. Second, while we focus on three core metrics (accessibility, coverage, and load balancing), the construction of new stations involves additional considerations like land and construction cost and local power grid capacity, which could be incorporated into our formulation. Third, the type of charging technology (e.g., level 1/2/3 chargers) could be incorporated into EV-Planner (e.g., by associating a vector with each cluster that represents how many chargers of each type are present in the corresponding charging station). However, this would require additional information regarding the breakdown of EVs based on compatible charger types. Fourth, accessibility in our work is quantified as the number of users who do not have access to a station within a specified distance. Socioeconomic and demographic considerations may also need to be incorporated in order to adopt such methods in practice. Finally, we focus on a static view of EV locations in order to determine demand for charging stations. Incorporating the dynamic nature of EV locations and diurnal patterns (*e.g*., times of peak load may coincide with commuting hours) would further improve the quality of the produced solutions, while of course making the problem more complex from a computational perspective.

## Data Availability

Publicly available datasets were analyzed in this study. This data can be found at: EV Registration Data (AtlasEVHub): https://www.atlasevhub.com/materials/state-ev-registration-data, EV Charging Station Data (U.S. DoE): https://afdc.energy.gov/data, and Population Density Data (Kontur): https://www.kontur.io/datasets/population-dataset/.
